# Short-term outcome associated with disease severity and electrolyte abnormalities among critically ill children with acute kidney injury

**DOI:** 10.1186/s12882-019-1278-1

**Published:** 2019-03-12

**Authors:** Osama Y. Safder, Khalid A. Alhasan, Mohamed A. Shalaby, Norah Khathlan, Suleman A. Al Rezgan, Amr S. Albanna, Jameela A. Kari

**Affiliations:** 1 Pediatric Nephrology Center of Excellence, Pediatric Department, Faculty of Medicine, King Abdulaziz University, PO Box 80215, Jeddah, 21589 Kingdom of Saudi Arabia; 2 Pediatric Intensive Care Unit, Department of Pediatrics, King Abdulaziz University, Jeddah, Kingdom of Saudi Arabia; 3 Pediatrics Department, College of Medicine. King Saud University, Riyadh, Kingdom of Saudi Arabia; 4 King Fahad Armed Forced Hospital, Jeddah, Kingdom of Saudi Arabia; 5 King Abdullah International Medical Research Center, King Saud Bin Abdulaziz University for Health Sciences, Jeddah, Kingdom of Saudi Arabia

**Keywords:** Acute kidney injury, Children, KDIGO, Mortality, Morbidity

## Abstract

**Background:**

Acute kidney injury (AKI) in critically ill children is associated with increased mortality and morbidity. In this study we evaluated the effect of AKI severity on the incidence of short-term mortality and morbidity.

**Methods:**

Multicenter prospective cohort study was conducted over two years period. We used the Kidney Disease Improving Global Outcomes (KDIGO) to diagnose and stage AKI.

**Results:**

A total of 511 out of 1367 included children (37.4%; 95% CI: 34.8–40.0) were diagnosed with AKI. They were categorized into three KDIGO stages: stage I (mild) in 47.5% (95% CI: 43.2–52.0), stage II (moderate) in 32.8% (95% CI: 28.8–37.1) and stage III (severe) in 19.7% (95% CI: 16.4–23.5). Stage II and III AKI had higher risk of mortality and longer length of stay (LOS) in hospital.

Children with stage III AKI were more likely to require mechanical ventilation, referral to pediatric nephrology and discharge with abnormal creatinine level (above 100 uml\L).

Hypervolemia, hypocalcemia, anemia, and acidosis were found to be independent risk factors of mortality.

**Conclusion:**

The extent of severity of AKI is directly associated with increased mortality, LOS and short-term morbidity.

## Background

Acute kidney injury (AKI) is associated with increased mortality, prolonged mechanical ventilation, and prolonged length of stay in intensive care units in both adults and children [1–3]. Staging of AKI provides important additional values for patient’s assessment and prediction of their prognosis. Studies on adults show linear association between the severity of AKI and disease outcomes [2]. The study of Assessment of Worldwide Acute Kidney Injury, Renal Angina, and Epidemiology (AWARE) indicates that AKI is common in critically ill children and young adults and is associated with poor outcomes, including increased mortality [1]. We have recently reported that AKI is common in children admitted to pediatric intensive care unit (PICU) and is also associated with higher mortality [4]. In addition, AKI is often associated with multiple complications including volume overload, metabolic acidosis and electrolyte disturbances such as hyperkalemia, hypocalcemia and hyperphosphatemia [5]. The relationship between electrolyte imbalance related to AKI and mortality has not been clearly determined.

In this study we investigated the short-term outcome associated with Kidney Disease Improving Global Outcomes (KDIGO) [6] staging and electrolyte abnormalities among pediatric patients admitted to PICU with AKI.

## Methods

A prospective cohort study was performed on critically ill children admitted to the PICU of three tertiary Care hospitals. The study duration was over two years period (March 2014 to February 2016). The Kidney Disease Improving Global Outcomes (KDIGO) definition was used to diagnose AKI [5].

All children admitted to PICU for at least 24 h were evaluated. For patient who required multiple PICU admissions, only the first admission was considered. Children were defined as aged 14 years old or younger.

We excluded neonates (defined as age less than 28 days), patients with evidence of preexisting chronic kidney disease (CKD) stage III –V, patients admitted electively for central line insertion and patients with insufficient data.

Estimated GFR (eGFRl) was calculated using the modified Schwartz formula [7].

To detect an expected 10% increase in mortality among critically ill patients who develop AKI, considering a baseline average mortality rate of around 10%, an estimated sample size of 438 (219 subjects with and without AKI) is required (power of 80% and alpha of 0.05). However, a larger sample size was planned in this multicenter study to increase the power for detecting factors that predict mortality.

### Definitions

We defined baseline creatinine as the last creatinine level performed within 6 months prior to PICU admission. For patients who were admitted for the first time with no previous creatinine, we estimated the average GFR based on their gender, age, and height.

Oliguria was defined as UOP of less than 0.5 ml\kg\hr. in children and less than 1 ml\kg\hr. in infants. Hypervolemia was defined as positive fluid balance more than 10% of dry weight.

Hyperkalemia was defined as serum potassium level more than 5.5 mmol\L. Hypocalcemia was defined as a serum calcium level less than 2 mmol\L. Hyperphosphatemia was defined as serum phosphate level more than 1.5 mmol\L. Acidosis was defined as pH less than 7.35 and bicarbonate level less than 22 mmol\L. Anemia was defined as reduction of HGB concentration two standard deviations below the mean, based on age-specific normals.

KDIGO consensus defines AKI as an increase in serum creatinine level to ≥0.3 mg\dl(≥26.5 mmol\l) within 48 h, increase in serum creatinine by ≥1.5 times from baseline, or decrease in urine volume to ≤0.5 ml\kg\hour. It classifies AKI into 3 stages, based on the magnitude of changes in serum creatinine and\or UOP levels, as follow: Stage(I) increase of serum creatinine to ≥0.3 mg\dl(≥26.5 mmol\l) or by 1.5 to 1.9 times from baseline and\or decrease in UOP to less than 0.5 ml\kg\hour for 6–12 h, Stage(II) increase of serum creatinine by 2 to 2.9 times from baseline and\or decrease in UOP to less than 0.5 ml\kg\hour for > 12 h, and stage(III) increase of serum creatinine to ≥353.6 mmol/L or by greater than 3 times from baseline, decrease in glomerular filtration rate (GFR) to less than 35 ml\min\1.73 m2, and\or decrease in UOP to less than 0.5 ml\kg\hour for > 24 h or anuria for > 12 h.

We used Pediatric Risk of Mortality (PRISM) Score version II, which includes 14 variables, to assess the relationship between physiological status and risk of mortality as one of morbidity indicators.

### Outcomes

The primary outcome measure was mortality, and the secondary outcomes were PICU length of stay (LOS), hospital LOS, hypertension, need to start renal replacement therapy and discharge with abnormal creatinine level, which were classified as measures of morbidity.

### Statistical analysis

We used STATA software (StataCorp. 2011: Release 12. College Station, TX: StaCorp LLC) for all analyses. The proportion and mean for categorical and continuous variables, respectively, were measured to describe the patients’ characteristics. The association between KDIGO stage and outcome measures was estimated using linear and logistic regression analyses for continuous and categorical outcome variables respectively. A multivariate regression analysis was performed to control for potential confounding factors, including baseline age, sex, and underlying diagnosis, which were determined based on a priori theoretical assumption using directed acyclic graphs. Statistical significance was determined with a *p*-value of 0.05, and data are presented with the 95% confidence intervals (CIs).

## Results

**Fig. 1:**
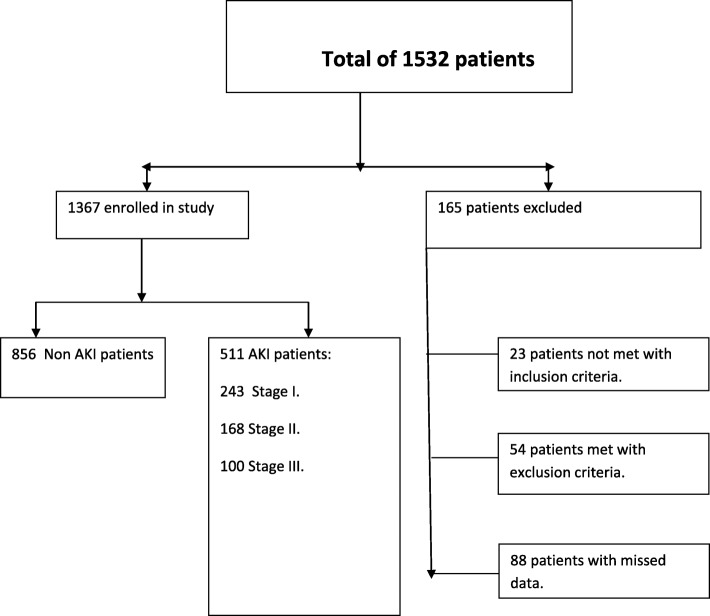
Study flow chart

One thousand five hundred thirty-two patients were assessed, of which1367 children were included as they fulfilled the inclusion criteria. We excluded 165 patients (77 children who did not meet the inclusion criteria and 88 patients because of missing data, Fig. 1). AKI based on KDIGO criteria was diagnosed in 511 (37.4%%; 95% CI: 34.8–40.0) of included patients. Based on KDIGO criteria, stage I (mild) was diagnosed in 47.5% (95% CI: 43.2–52.0) of all AKI patients, stage II (moderate) was diagnosed in 32.8% (95% CI: 28.8–37.1) and stage III (severe) was diagnosed in 19.7% (95% CI: 16.4–23.5).

Table 1 shows the baseline demographic and disease characteristics of patients with different AKI stages. Patients with severe AKI (Stage III) were having the highest creatinine and lowest GFR levels, higher PRISM score and low UOP recorded during PICU admission.

**Table 1:** Baseline Patients’ demographic and disease characteristics

	KDIGO I		KDIGO II		KDIGO III	
	Estimate	95% CI	Estimate	95% CI	Estimate	95% CI
Age (mean, months)	47.0	40.5–53.4	45.3	37.4–53.2	38.8	29.1–48.4
Female sex (%)	48.8	41.3–54.2	46.2	38.5–54.0	43.4	33.5–53.8
Saudi Nationality (%)	47.7	41.3–54.2	43.5	35.9–51.3	41.5	31.4–52.1
Creatinine (mean, μmol/L)	33.2	31.9–34.6	31.8	30.3–33.4	31.3	29.4–33.3
Highest Creatinine	56.2	54.3–58.2	80.4	76.2–84.6	270	218–321
GFR (mean, ml/min/1.73 m2)	112	106–117	108	101–114	108	99–116
Lowest GFR	67.5	64.4–70.5	46.2	42.9–49.4	18.3	16.1–20.5
Nephrology referral (%)	14.3	10.2–19.4	53.8	46.0–61.5	93.9	87.3–97.7
Need for ventilatory support (%)	53.7	47.2–60.1	62.1	54.4–69.5	85.9	77.4–92.0
PRISM (mean)	7.9	7.0–8.6	14.3	12.7–15.8	27.7	24.5–30.9

The main underlying etiology of AKI was related to cardiac conditions (cardiac surgery in and heart failure) followed by sepsis (Table 2).

**Table 2:** Underlying etiology of acute kidney injury among patients admitted to pediatric intensive care unit

Underlying Disease Category	KDIGO 1		KDIGO 2		KDIGO 3	
	%	95% CI	%	95% CI	%	95% CI
1. Sepsis	32.1	26.2–38.4	32.7	25.7–40.4	40.4	31.3–22.4
2. Hypoxia	18.3	13.7–23.8	18.5	12.9–25.2	10.1	5.0–17.8
3. Heart failure	6.3	3.5–10.1	8.3	4.6–13.6	5.1	1.7–111.4
4. Obstructive uropathy	1.7	0.5–4.2	1.8	0.4–5.1	6.1	2.3–12.7
5. Dehydration	2.9	1.2–5.9	6.6	3.3–11.4	0	0–3.7
6. Glomerulonephritis	1.7	0.5–4.2	1.8	0.4–5.1	8	3.6–15.3
7. Toxic	0.4	0.01–2.3	1.2	0.1–4.2	2	0.3–7.1
8. MOSF	3.3	1.5–6.5	6.6	3.3–11.4	19.2	12.0–28.3
9. TLS	2.9	1.2–5.9	0.6	0.02–3.3	4	1.1–10.0
10. Renal neoplasm	0.4	0.01–2.3	0.6	0.02–3.3	0	0–3.7
11. HUS	0	0–1.5	0	0–2.2	1	0.03–5.5
12. Post-cardiac^a^	30	24.3–36.2	21.4	15.5–28.4	13.1	7.2–21.4

Abbreviations: *KDIGO* Kidney Disease Improving Global Outcomes, *MOSF* Multi-Organ System Failure, *TLS* Tumor Lysis Syndrome, *HUS* Hemolytic Uremic Syndrome

Notes: ^a^post-cardiac surgery

Patients with different categories of AKI had higher risk of mortality and higher PRISM score in comparison with non-AKI patients, and this risk is increased with increasing severity of AKI. Stage 1 AKI carried double the risk of mortality (OR: 2.54, 95% CI: 1.39–4.62, *P* value: 0.002) as compared to non-AKI, with a mean PRISM score of 7.9 (SD: 6.5, 95% CI: 7.0–8.6). While in-hospital mortality was even higher with stage II (OR: 6.3, 95% CI: 3.65–10.96, *P* value < 0.001) and stage III (OR: 23.9, 95% CI: 13.4–42.4, *P* value < 0.001), with mean PRISM scores of 14.3 (SD: 10.3, 95% CI: 12.7–15.8) and 27.7 (SD: 16.0, 95% CI: 24.5–30.9), respectively.

As predictors of renal morbidity, all these stages of AKI were associated with significantly higher risk of hypertension during admission (OR: 2.68 [95% CI: 1.66–4.34], 4.10 [95% CI: 2.52–6.67], and 8.12 [95% CI: 4.79–13.8] respectively).

Children with severe renal injury (stage III KDIGO) had longer length of stay both in PICU and hospital, which was statistically significant (Table 3).

**Table 3:** Clinical outcomes associated with stages of severity of acute kidney injury

Characteristics	KDIGO 1		KDIGO 2	KDIGO 3		
	OR* (95% CI)	*P* value	OR* (95% CI)	*P* value	OR* (95% CI)	*P* value
In-hospital mortality	2.54 (1.39–4.62)	0.002	6.33 (3.65–10.96)	< 0.001	23.9 (13.4–42.4)	< 0.001
Hypertension	2.68 (1.66–4.34)	< 0.001	4.10 (2.52–6.67)	< 0.001	8.12 (4.79–13.8)	< 0.001
	Coefficient* (95% CI)	*P* value	Coefficient* (95% CI)	*P* value	Coefficient* (95% CI)	*P* value
LOS in PICU (days)	7.6 (6.0–9.1)	< 0.001	11.6 (9.8–13.4)	< 0.001	12.2 (9.9–14.4)	< 0.001
LOS in hospital (days)	8.0 (5.3–10.7)	< 0.001	15.0 (11.9–18.1)	< 0.001	15.5 (11.5–19.4)	< 0.001

Abbreviations: *KDIGO* Kidney Disease Improving Global Outcomes, *LOS* length of stay, *PICU* pediatric intensive care unit

*Referenced to values of patients with normal renal function and adjusted for age, sex, and underlying diagnosis

The proportion of patients with stage III AKI who required mechanical ventilation and referral to pediatric nephrology team were 85.9% (95% CI: 77.4–92.0) and 93.9% (95% CI: 87.3–97.7), respectively, which were higher than the proportions with stage II (62.1% [95% CI: 54.4–69.5] and 53.8% [95% CI: 46.0–61.5], respectively) and stage I (53.7% [95% CI: 47.2–60.1] and 14.3% [95% CI: 10.2–19.4], respectively).

GFR level during admission was not an important predictor of in-hospital mortality (OR: 0.97, 95% CI: 0.96–0.98); however, oliguria was an important predictor (OR: 2.5, 95% CI: 1.61–4.0) but not independently. Other predictors that are independently associated with mortality are hypervolemia, hypercalcemia, anemia and acidosis (Table 4).

**Table 4:** Clinical and laboratory features of renal impairment that are predictor of mortality

Characteristics	Univariant			Multivariant		
	OR	95% CI	*P* value	OR	95% CI	*P* value
Hypertension	2.4	1.5–3.8	< 0.001	0.74	0.38–1.44	0.37
Worse GFR level	0.97	0.96–.98	< 0.001	0.997	0.98–1.01	0.68
Oliguria	2.5	1.61–4.0	< 0.001	0.99	0.54–1.81	0.97
Hypervolemia	24.4	15.1–39.4	< 0.001	5.3	2.6–10.9	< 0.001
Hypernatremia	3.1	1.9–5.0	< 0.001	1.9	0.98–3.6	0.57
Hyponatremia	1.7	0.84–3.4	0.141	1.04	0.45–2.4	0.92
Hyperkalemia	3.5	2.2–5.5	< 0.001	1.16	0.64–2.1	0.63
Hypocalcaemia	5.8	3.5–9.5	< 0.001	2.02	1.06–3.8	0.03
Hyperphosphatemia	2.5	1.6–4.0	< 0.001	0.7	0.38–1.32	0.27
Anemia	11.3	6.1–21.0	< 0.001	4.5	2.1–9.4	< 0.001
Acidosis	4.6	2.8–7.4	< 0.001	1.85	1.0–3.4	0.05

Twenty nine percent (95% CI: 20.4–38.9) of patients with severe AKI (Stage III) were discharged with abnormal creatinine level (more than 100 uml\L). In comparison, 5% (95% CI: 2.5–9.9) of patients with moderate form AKI (Stage II) and none of patients with mild AKI (Stage I) were discharged with high creatinine level.

Renal replacement therapy (RRT) was used in 11.4% (95% CI: 8.7–14.4) of patients with AKI. The most commonly used RRT modality was peritoneal dialysis (PD) (70.7, 95% CI: 57.3–81.8), followed by continuous renal replacement therapy (CRRT) (17.2, 95% CI: 8.6–29.4) and hemodialysis (HD) (8.6, 95% CI: 2.9–19.0). Two (3, 95% CI: 0.4–11.9) patients underwent both PD and HD.

As shown in Table 4, fluid and electrolyte changes that independently predict mortality were hypervolemia, hypocalcemia, anemia, and acidosis.

## Discussion

In this multicenter study, we found that even a small rising in serum creatinine level and mild AKI are associated with increased mortality rate and length of stay in PICU and hospital. This is similar to previous studies [5, 8], such as Bailey et al. study which reported higher mortality rate in AKI patients as compared to patients with normal kidney function (29.6% vs. 2.3%, *p* < .001) [5]. We also observed that severe AKI (stage 3) is associated with higher mortality rate and higher PRISM II score, which is well known validated factor for predicting mortality rate among children admitted to PICU.

In our study, AKI was associated with increased length of hospital stay and the need for ventilatory support, which was more frequent with severe AKI. This could be explained by the fact that oliguria is more common in severe AKI which could lead to volume overload and therefore the need for mechanical ventilation. Based on previous studies, the need for mechanical ventilation and initiation of renal replacement therapy are both independent risk factors for mortality in sick children with AKI [9].

We observed high incidence of AKI in children admitted to PICU (37.4%). This is similar to previous reports [1, 10, 11], including a recent large prospective multicenter study of 32 PICUs where AKI, using KDIGO definition, was diagnosed in 26.9% of total admissions [13]. However, the reported incidence of AKI among children and young adults varies widely from 5 to 82% and this might be as a result from co-existing conditions, severity of underlying etiology and different used definitions [1, 12, 13].

The majority of patients in our study who developed AKI were young children aged less than 5 years. This observation is similar to other studies [14, 15] indicating that younger children are more prone to AKI and mandates the need for a closer monitoring of serum creatinine and urine output.

The major underlying etiology in our cohort were cardiac conditions such as post cardiac surgery or heart failure. Risk factors which increase the incidence of post cardiac AKI include preoperative anemia, low perioperative GFR (60 ml/1.73 m2/min) and the use if intra-operative balloon pump [16]. Sepsis was also a major contributing factor for AKI in our cohort. This is a similar to a previous report in which infection was found to be the main underlying cause of AKI [17].

Hypervolemia was associated with increased mortality in our study. This is in line with previous report by Alkandari et al. which indicates that the development of hypervolemia was associated with prolongation of use of mechanical ventilator and longer duration of hospital stay [3].

Anemia is common in AKI and is usually multifactorial in origin including decreased erythropoiesis, bleeding and reduced red blood cells survival. We found that the presence of anemia is associated with poor outcome in sick children with AKI. Kenyber et al., reported that anemia and the need for blood transfusion are associated with increased mortality rate, prolonged use of vasoactive medications and prolonged PICU stay [18].

Acidosis was also a risk factor for higher mortality rate in children with AKI. This finding is similar to previous report of 123 children with AKI where acidosis and elevated serum lactate were associated with increased mortality [19].

Hypocalcemia is another risk factor for death in sick children with AKI in our study. Singhi at al reported higher mortality in AKI children with hypocalcemia (28.3%) compared with normo-calcemic (7.5%) patients (*p* < 0.05) [20]. However another study from Korea showed that hyperphosphatemia is a potential marker that can reflect disease severity and predict mortality in severe AKI patients receiving CRRT [21].

These findings may suggest that hypocalcemia, acidosis and anemia can potentially be used as markers to evaluate the severity of AKI.

We observed abnormal high creatinine at discharge in one third of stage III AKI, compared to 5.3% of stage II AKI and none of those with stage I. There are paucity of studies evaluating renal recovery in pediatric patients. Studies on adults, however, showed that the rate of renal recovery varied from 36 to 68% [22, 23]. Children could have greater renal reserve and higher chance of recovery compared with adult [1]. We have previously reported a cosidrable percentage of children with evidence of chronic kidney disease following an episode of AKI [24].

Our study has several limitations as we have assessed AKI only in critically ill children admitted to PICU and we did not assess AKI in patients presented to emergency department or patients admitted to pediatric medical ward. Therefore, we did not address all types of community acquired AKI. On the other hand, our study has several strength points as it is a prospective multicenter study that involves a large number of patients with a variety of multiple etiological factors. This provide adequate power to study the relationship between different exposures and outcomes.

## Conclusions

AKI is common in critically ill children admitted to PICU. The degree of severity of AKI is associated with a higher risk of death, longer hospital stay and more need for mechanical ventilator support. The presence of anemia, hypocalcemia and acidosis are associated with higher mortality among critically ill children with AKI.

## Abbreviations

AKI: Acute kidney injury
AWARE: Assessment of Worldwide Acute Kidney Injury, Renal Angina, and Epidemiology
CKD: Chronic kidney disease
CRRT: Continuous Renal Replacement Therapy
GFR: Glomerular filtration rate
HD: Hemodialysis
ICU: Intensive care unit
KDIGO: Kidney disease improving global outcome
KDIGO: Kidney Disease Improving Global Outcomes
LOS: Length of stay
PD: Peritoneal dialysis
PICU: Pediatric intensive care unit
PRISM: Pediatric Risk of Mortality
UOP: Urine output
