# Correction to: Short-term outcome associated with disease severity and electrolyte abnormalities among critically ill children with acute kidney injury

**DOI:** 10.1186/s12882-020-01790-0

**Published:** 2020-04-16

**Authors:** Osama Y. Safdar, Khalid A. Alhasan, Mohamed A. Shalaby, Norah Khathlan, Suleman A. Al Rezgan, Amr S. Albanna, Jameela A. Kari

**Affiliations:** 1 Pediatric Nephrology Center of Excellence, Pediatric Department, Faculty of Medicine, King Abdulaziz University, PO Box 80215, Jeddah, 21589 Kingdom of Saudi Arabia; 2 Pediatric Intensive Care Unit, Department of Pediatrics, King Abdulaziz University, Jeddah, Kingdom of Saudi Arabia; 3 Pediatrics Department, College of Medicine, King Saud University, Riyadh, Kingdom of Saudi Arabia; 4 King Fahad Armed Forced Hospital, Jeddah, Kingdom of Saudi Arabia; 5 King Abdullah International Medical Research Center, King Saud Bin Abdulaziz University for Health Sciences, Jeddah, Kingdom of Saudi Arabia

**Correction to: BMC Nephrology (2019) 20:89.**

**https://doi.org/10.1186/s12882-019-1278-1**

Following publication of the original article [1], we have been notified that there is a typo in the name of the first author.

Incorrect: Osama Y Safder.

Correct: Osama Y Safdar.
